# Probiotic-Induced Gut Microbiota Modulation: A Comparative Analysis Using 16S rRNA V3–V4 and Targeted Sequencing

**DOI:** 10.3390/microorganisms14051035

**Published:** 2026-05-01

**Authors:** Han Lee, Gaeun Kim, Jungeun Kim, OneZoong Kim, Sung-Hee Jung, Sunghee Hyun, Chang Seok Oh

**Affiliations:** 1 Department of Biomedical Laboratory Science, Graduate School, Eulji University, Uijeongbu 11759, Republic of Korea; barby112@naver.com (H.L.); wendystar@naver.com (G.K.); chohwa0911@naver.com (J.K.); 2 Department of Internal Medicine, Yeouido St. Mary’s Hospital, The Catholic University of Korea, Seoul 07345, Republic of Korea; biblian1@outlook.com; 3 Department of Gastroenterology, Daejeon Eulji University Hospital, Eulji University, Daejeon 35233, Republic of Korea; jsh@eulji.ac.kr; 4 Department of Senior Healthcare, Graduate School, Eulji University, Uijeongbu 11759, Republic of Korea; 5 Department of Mortuary Science, College of Bio-Convergence, Eulji University, Seongnam 13135, Republic of Korea

**Keywords:** 16S rRNA, gut microbiota, probiotics, target species

## Abstract

Standard 16S rRNA V3–V4 sequencing encounters primer mismatch issues and insufficient taxonomic resolution, hindering the accurate quantification of specific, low-abundance taxa, such as administered probiotic strains. Therefore, we empirically compared outcomes between the standard V3–V4 method and high-resolution targeted species sequencing (TSS) to assess bias and establish reliability metrics for probiotic efficacy assessments. A longitudinal pilot study was conducted over nine weeks in older participants receiving synbiotic supplementation; their fecal samples were collected and analyzed. V3–V4 analysis successfully captured a significant transient reduction in alpha-diversity with multidirectional genus-level fluctuations. However, taxonomic overlap between these two methods was high at the phylum level and sharply declined to 6.7% at the species level. Notably, compared with V3–V4 sequencing, TSS could successfully quantify the abundance of administered *Bifidobacterium animalis*. This study empirically demonstrated that 16S rRNA V3–V4 sequencing introduces substantial quantitative bias, limiting its suitability for monitoring specific probiotic strains and compromising the reliability of clinical efficacy assessments. Therefore, we recommend a dual-sequencing framework that integrates the broad ecological screening capabilities of V3–V4 with the precise species-level quantification of TSS to establish the necessary scientific rigor for assessing probiotic efficacy.

## 1. Introduction

The gut microbiota is a important factor in host health [1], and disruptions in its diversity (dysbiosis) during aging strongly correlates with conditions such as inflammation and functional decline [2,3]. This dysbiosis is associated with altered immune responses that can lead to various disease states [4]. Probiotic intervention, which involves the administration of beneficial live microorganisms, is a key strategy used for restoring microbial balance and alleviate age-related symptoms [5,6,7,8]. Therefore, accurately monitoring the fate and ecological impact of these probiotic strains within the complex microbial community of the host is essential for validating their efficacy [9].

Traditionally, microbial community profiling has heavily relied on 16S rRNA gene sequencing of specific hypervariable regions, such as the V3–V4 region [10,11]. Although this method is cost-effective and efficient for characterizing the overall community structure across large cohorts, it lacks sufficient species-level discriminatory power owing to the high sequence homology across bacterial taxa [10,12]. This presents significant challenges for evaluating targeted interventions, such as probiotics, specifically including primer mismatch-induced amplification bias and inadequate taxonomic resolution, which hinder the accurate quantification of specific, low-abundance yet functionally crucial taxa (including administered probiotic species) [13,14,15]. Indeed, in our V3–V4 analysis of older adults following probiotic intake, we observed complex, multidirectional remodeling of the gut ecosystem, characterized by both decreases and compensatory increases in functional taxa (e.g., *Faecalibacterium* and *Blautia*). These heterogeneous and sometimes conflicting community-level shifts highlight the limitations of relying solely on a single, broad-spectrum sequencing method to elucidate the precise mechanisms underlying probiotic action [16].

To overcome the well-recognized limitations in species-level resolution and quantification bias inherent in 16S rRNA V3–V4 sequencing, a high-resolution, strain-specific approach is required [12,17]. Targeted species sequencing (TSS), which employs species-specific primers that span multiple hypervariable regions, provides markedly improved accuracy for detecting and quantifying low-abundance bacterial strains [16,18,19].

Therefore, the aim of this study was to directly and empirically compare the outcomes of two distinct microbiome profiling methodologies: standard 16S rRNA V3–V4 sequencing and the high-resolution TS approach. By performing a comparative analysis of fecal samples collected during synbiotic intervention in older adults, this study aimed to precisely identify and quantify discrepancies between the two sequencing platforms. This comparison is critical for determining whether the V3–V4 method misrepresents or fails to detect key functional taxa (including the administered probiotic species) and for establishing the need for a dual-sequencing framework to achieve accurate and sensitive evaluations of probiotic efficacy in future clinical studies.

## 2. Materials and Methods

### 2.1. Study Design and Sample Collection

This study was conducted with a cohort of 20 middle-aged to older individuals (aged 60–70 years), predominantly female (85%), who were recruited from a community institution in Seongnam, Republic of Korea (Table 1). Participants were confirmed free of specific underlying diseases. The study spanned nine weeks and used a time-series design across three defined time points: time points 1 (T1; week 3), 2 (T2; week 6), and 3 (T3; week 9). The initial three weeks served as the run-in period. The intervention period was defined as the three weeks occurring between T1 and T2, during which participants received the commercial synbiotic supplement daily. The Control group received no synbiotic intervention. Specifically, the Control group consisted of four individuals who consumed the same beverage that the treatment group did but received a placebo (a drink without the bacterial strain). Participants were assigned to the Control and Treatment groups via simple random assignment. The subsequent 3-week period (T2–T3) served as the control washout period. Sixty fecal samples (*n* = 20 individuals × 3 time points) were collected for microbial analysis. This study was approved by the Eulji University Internal Review Board (IRB No. EUIRB 2021-008), and written informed consent obtained from all participants prior to the study.

The participants were issued sterile collection containers and instructed to collect a minimum of 10 g fresh stool immediately before their visit. Stool samples were stored at 4 °C in a domestic refrigerator before delivery. Upon arrival at the laboratory, the samples were kept at 4 °C, and DNA extraction initiated within 2 h of receipt.

### 2.2. Intake Supplements

The intervention used a commercially available synbiotic formulation (MPRO3; hy Co., Ltd., Yongin-si, Gyeonggi-do, Republic of Korea). The daily dose provided a total bacterial count of 10 × 10^9^ colony-forming units (CFUs) delivered in a composite package that included two capsules (130 mg/capsule) and 130 mL of a liquid solution, with the capsule and liquid components designed in a respective 1:2 ratio. The precise probiotic composition consisted of three distinct strains: *Bifidobacterium animalis* subsp. *lactis* HY8002 (5.0 × 10^9^ CFU), *Lactobacillus casei* HY2782 (2.5 × 10^9^ CFU), and *L. plantarum* HY7712 (2.5 × 10^9^ CFU). The 130 mL solution served as the prebiotic component, containing various forms of dietary fiber (including polydextrose, chicory dietary fiber, lactulose, and wheat dietary fiber) and several functional oligosaccharides (such as fructooligosaccharides, isomaltooligosaccharides, and xylooligosaccharides).

### 2.3. DNA Extraction and PCR Amplification

Genomic DNA was extracted from fecal samples using the QIAamp PowerFecal Pro DNA Kit (Qiagen, Hilden, Germany), following the manufacturer’s protocol. The process commenced by accurately measuring a 250 mg aliquot of the fecal material and transferring it into a dry bead tube provided by the kit. Subsequently, 800 L C1 Lysis Solution was added, and the mixture mechanically vortexed for 10 min. Following agitation and centrifugation, the supernatant was carefully separated, 200 L CD2 solution added, and the sample processed further. The recovered supernatant, excluding the residual pellet, was then mixed with 600 L CD3 solution. This mixture was loaded onto the spin column in two sequential applications (650 L each), separated by centrifugation steps. The column was washed with 500 L EA and 500 L C5 solutions, each followed by centrifugation. The purified DNA was eluted by applying 65 L C6 elution solution, whereafter the sample was centrifuged and the final solution collected. All extracted DNA samples were stored at −80 °C until library preparation and sequencing were initiated.

### 2.4. 16S rRNA Amplicon and Targeted Species Sequencing

The extracted DNA served as the template for PCR amplification targeting the V3–V4 hypervariable region of bacterial 16S rRNA genes. Amplification employed the following universal primers: 341F (5′-CCTACGGGNGGCWGCAG-3′), which contained a sample-specific 6–8 bp tag sequence, and 805R (5′-GACTACHGGGTATCTAATCC-3′). PCR was performed using the Platinum PCR SuperMix High Fidelity system (Thermo Fisher Scientific, Waltham, MA, USA). Each 27 µL final reaction volume contained 2.5 ng template DNA and primers at a final concentration of 50 nM. The thermal cycling protocol was structured as follows: initial denaturation at 94 °C for 3 min, followed by 30 cycles of denaturation at 94 °C for 30 s, annealing at 50 °C for 30 s, and extension at 72 °C for 30 s. The resulting amplicon libraries were further purified using the Agencourt AMPure XP DNA Purification Kit (Beckman Coulter, Brea, CA, USA) to eliminate residual primer dimers and other contaminants, following the manufacturer’s instructions. The purified samples were eluted in 15 µL low-EDTA Tris-EDTA buffer.

DNA concentration, purity, and amplicon library concentrations were precisely measured using the dsDNA HS (High Sensitivity) Assay Kit on a Qubit 4 Fluorometer (Thermo Fisher Scientific). Fragment size distribution and overall quality of the pooled DNA libraries were subsequently verified using the Agilent 2100 Bioanalyzer system (Agilent Technologies, Palo Alto, CA, USA). The enriched libraries were loaded onto an Ion 530 Chip Kit (Thermo Fisher Scientific). Sequencing was then performed using the Ion GeneStudio S5 next-generation sequencing system (Thermo Fisher Scientific) to generate paired-end reads, following the standard operational procedures of the platform.

TSS was performed using an Ion AmpliSeq technology-based assay to ensure accurate species-level resolution (Ion AmpliSeq Microbiome Health Research Kit, Thermo Fisher Scientific). This assay incorporates a comprehensive 16S design covering eight hypervariable regions, along with highly species-specific primers targeting 73 bacterial species (Supplementary Data S1), including the administered probiotic strains. The TSS method has been validated to provide 100% sensitivity and specificity at the species level, demonstrating superior quantitative accuracy (Spearman’s rho: 0.90–0.99) compared with that of standard 16S methods.

### 2.5. Data Processing

Raw 16S rRNA sequence data, provided in FASTQ format, were initially generated and managed using Torrent Suite Software (v.5.14.1.1; Thermo Fisher Scientific). Amplicon sequence variant (ASV) inference and downstream bioinformatics processing were primarily conducted using the Quantitative Insights into Microbial Ecology (QIIME 2 v.2.0) software environment following the DADA2 pipeline. A total of 1,211,742 raw read counts were acquired across all samples, with an average of 4627 counts/sample. Prior to ASV inference, sequences underwent quality filtering and trimming based on the sample origin due to varying read quality profiles: sequences were truncated to 418 bp, and the initial 47 bp containing low-quality data removed. Chimeric sequences were also addressed at the beginning of each read. The assembled reads were subsequently demultiplexed and processed through the DADA2 pipeline using default parameters. Taxonomic identification of the resulting ASVs was performed using the EzBio-classifier database. For comparative analysis, clusters were generated based on a 97% sequence similarity threshold, with a focus on downstream analysis centered on the genus level of classification. Reads generated via the TS assay were separately processed using the dedicated Ion Reporter solution workflow, which leveraged species-specific primer information for accurate taxonomic classification down to the species level.

### 2.6. Statistical Analysis

The generated ASVs were used as inputs for microbial diversity assessments (alpha- and beta-diversity) conducted within the QIIME 2 framework. To evaluate the alpha-diversity within sample communities, standardized metrics, including taxonomic richness (observed features), were calculated after the samples had been rarefied to an equal sequencing depth. Differences in alpha-diversity between comparison groups were statistically assessed using the nonparametric Mann–Whitney U test. For beta-diversity assessment, the unweighted UniFrac distance metric was employed to quantify the phylogenetic dissimilarity in bacterial community structure among samples, with differences in community structure between groups analyzed for statistical significance using the Analysis of Similarities (ANOSIM) test. Furthermore, to compare the abundance profiles and highlight taxonomic distinctions between microbial communities, a tree analysis approach was used. This method leveraged the hierarchical structure of taxonomic classification, incorporating both quantitative metrics (based on median abundance) and statistical evaluations (using the nonparametric Wilcoxon rank-sum test). A focused analysis of variance (ANOVA) with Bonferroni post hoc analysis was performed to assess quantitative agreement between the two platforms. All statistical analyses were supplemented and visualized using GraphPad Prism (v.9.3.0; GraphPad Software, Boston, MA, USA) and SPSS Statistics (v.20.0; IBM, Armonk, NY, USA), with differences considered statistically significant at *p*-values < 0.05.

## 3. Results

### 3.1. 16S rRNA V3–V4 Sequencing Analysis of the Gut Microbiota

#### 3.1.1. Overall Community Composition and Relative Abundance

**Figure 1:**
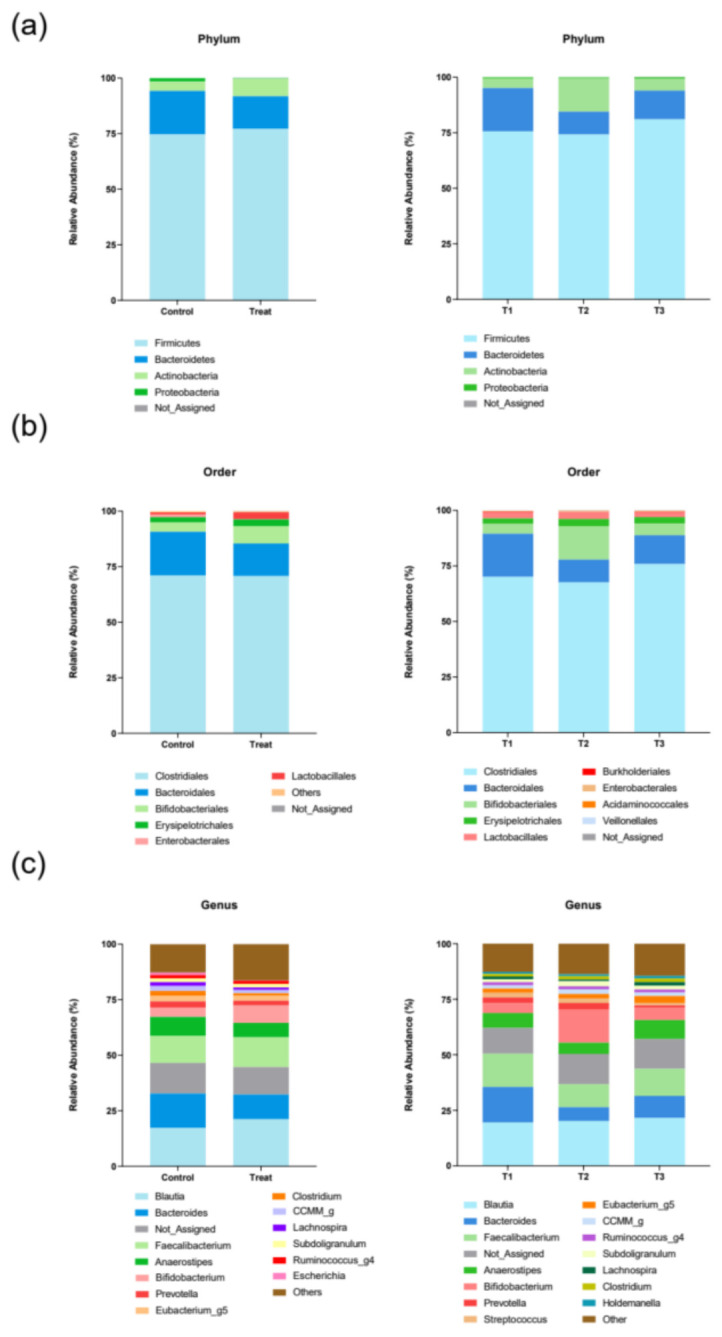
Relative abundance profiles of the major microbial taxa are shown for different groups (Control vs. Treatment) and across three time points (T1–T3). Compositional differences in the microbial communities based on phylogenetic classification are illustrated at the phylum (**a**), order (**b**), and genus (**c**) taxonomic levels. Abbreviation: T, time point.

Microbial community analysis based on 16S rRNA V3–V4 sequencing revealed the overall compositional gut microbiota profile of the study participants (Figure 1). The mean relative abundance profiles for the Control (no synbiotic intervention) and Treatment groups were largely similar, suggesting no significant baseline compositional differences in the major phyla detected.

The microbial community was primarily dominated by Firmicutes and Bacteroidetes, both accounting for approximately 90% of the total relative abundance observed across all time points. At baseline (T1), the abundance of Firmicutes was 75.6% and that of Bacteroidetes 19.4%. The phylum Actinobacteria exhibited the most significant fluctuation over time; its abundance increased from 4.3% at T1 to a peak of 15.0% at T2 (end of the intervention) before decreasing to 5.4% at T3. Conversely, Bacteroidetes abundance decreased from 19.4% at T1 to 10.2% at T2. Firmicutes abundance remained relatively stable between T1 (75.6%) and T2 (74.1%) but increased to 81.0% at T3 (Figure 1a). At the order level, Clostridiales (under Firmicutes) abundance slightly decreased from 70.0% (T1) to 67.6% (T2) before increasing to 75.8% (T3). Consistent with the phylum-level changes observed, Bifidobacteriales (under Actinobacteria) abundance dramatically increased from 4.3% at T1 to 15.0% at T2, while that of Bacteroidales simultaneously decreased from 19.4% to 10.2% over the same period (Figure 1b). Genus-level analysis corroborated the dynamics observed at higher taxonomic levels (Figure 1c). The genus of the administered probiotic strain, *Bifidobacterium*, showed the most pronounced temporal change, significantly increasing from 4.36% at T1 to 15.01% at T2 (nearly 3.4-fold increase) before dropping to 5.41% at T3. Concurrently, the relative abundances of two major short-chain fatty acid (SCFA)-producing genera decreased at T2: *Bacteroides* decreased from 15.98% (T1) to 6.23% (T2) and *Faecalibacterium* decreased from 14.97% (T1) to 10.32% (T2). Conversely, *Blautia* maintained a stable, high abundance, slightly increasing from 19.52% at T1 to 20.16% at T2 and 21.56% at T3.

#### 3.1.2. Alpha- and Beta-Diversity Assessments

**Figure 2:**
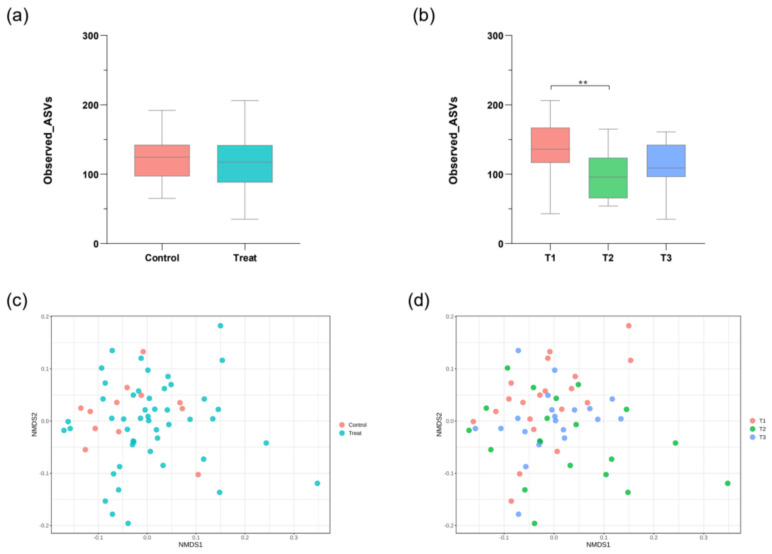
Phylogenetic and compositional differences of microbial communities are presented using alpha- and beta-diversity metrics. (**a**,**b**) Alpha-diversity analysis: Box plots showing alpha-diversity (within-sample diversity, e.g., observed amplicon sequence variants [ASVs]) comparing different groups. Statistical differences in alpha-diversity were primarily assessed using the Kruskal–Wallis test for overall group comparison, followed by Mann–Whitney U tests for pairwise comparisons. Box plots illustrate the 25th–75th percentile range, with the black horizontal line indicating the 50th percentile (median). (**c**,**d**) Beta-diversity analysis: Nonmetric multidimensional scaling (NMDS) plots visually represent the structural dissimilarity between groups, as quantified using the Weighted UniFrac distance metric. Statistical differences between communities were analyzed using Analysis of Similarities. Statistical notation: *p*-values for the Mann–Whitney U test (for pairwise alpha-diversity comparisons) are indicated in the figure: ** *p* < 0.01. Abbreviation: T, time point.

Taxonomic richness, quantified using the Observed ASVs metric, was assessed across both general treatment groups and at specific time points (Figure 2a,b). No statistically significant differences in alpha-diversity between the general Control and Treatment groups were observed. However, analysis across time points revealed significant changes: alpha-diversity decreased during the intervention period, with the difference between T1 (baseline) and T2 (end of intervention) being statistically significant (*p* = 0.013; Kruskal–Wallis test). No other significant pairwise differences were observed.

Community structural differences were evaluated using the Weighted UniFrac distance metric and visualized via nonmetric multidimensional scaling (NMDS) plots (Figure 2c,d). Comparisons between the Control and Treatment groups showed that the overall microbial community structures were not significantly separated (ANOSIM test: R = −0.55515, *p* = 0.706). In contrast, community structures assessed across the three time points within the Treatment group showed a statistically significant separation (ANOSIM test: R = 0.062287, *p* = 0.009). Despite this statistical significance, the NMDS plots demonstrated high inter-individual variability, with extensive overlap observed among the T1–T3 sample distributions.

#### 3.1.3. Complex and Unresolved Taxonomic Dynamics

**Figure 3:**
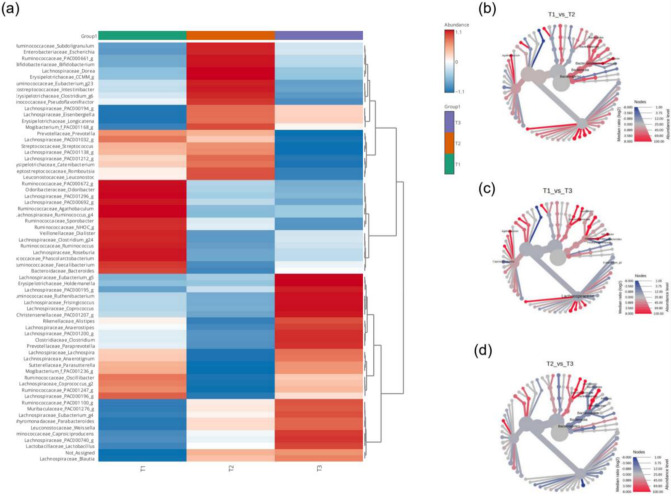
Hierarchical clustering heatmap of the gut microbiota composition across three time points (**a**). The heatmap depicts the relative abundance of dominant gut microbial genera across three time points: before (T1), during (T2), and after (T3) synbiotic intake. Colors represent z-score-normalized abundance values, where red indicates increased abundance, blue indicates decreased abundance, and white represents mean abundance levels. (**b**–**d**) Heat tree plots displaying taxonomic hierarchies with node colors representing relative enrichment (red: first group, blue: second group) based on median abundance, using the Wilcoxon rank-sum test for significance. Node sizes reflect the relative abundance, highlighting key taxa contributing to the compositional differences between groups. Abbreviation: T, time point.

The 16S rRNA V3–V4 sequencing analysis captured complex and multidirectional fluctuations in gut microbiota at the genus level following probiotic administration. The hierarchical clustering heatmap (Figure 3a) demonstrated distinct genus-relative abundance patterns at T2 compared with those at T1 and T3, suggesting a broad ecological reorganization. ANOVA and Bonferroni post hoc results (*p* < 0.05) confirmed statistically significant abundance changes in at least nine major functional taxa across the measured time points. For example, significant shifts were observed in *Bacteroides* (*p* = 0.001), *Roseburia* (*p* = 0.004), and *Odoribacter* (*p* = 0.005) during the T1 vs. T2 comparison. The heat tree plots (Figure 3b–d), based on the Wilcoxon rank-sum test, further validated these hierarchical changes; the abundances of *Bacteroides* (*p* = 0.001593) and Bacteroidales (*p* = 0.008712) significantly decreased between T1 and T2, indicating a significant competitive response from the Bacteroidetes phylum. Furthermore, V3–V4-based functional prediction analysis revealed significant fluctuations in the Kyoto Encyclopedia of Genes and Genomes pathways between T1 and T2 (Table 2). Notably, the predicted abundance of the butanoate metabolism pathway significantly increased from 0.0884 at T1 to 0.4658 at T2 (*p* = 0.0172), suggesting a potential enhancement in SCFA production capability during the intervention period. Conversely, abundance of the ko00604 glycosphingolipid biosynthesis pathway significantly decreased from 0.0566 at T1 to 0.0404 at T2 (*p* = 0.0063), and that of riboflavin metabolism decreased from 0.3653 to 0.3026 (*p* = 0.0035). While the V3–V4 data successfully captured this broad complexity and functional perturbation, inherent limitations of the method at the species level precludes objective quantitative validation of whether the observed increase in butanoate metabolism and taxonomic shifts were directly driven by successful colonization of the administered probiotic strain. To overcome this fundamental limitation of V3–V4 sequencing data and accurately verify the fate of the probiotic strain, we performed high-resolution TSS analysis, as described in Section 3.2.

### 3.2. Empirical Validation of Sequencing Discrepancy and Species Fidelity

**Figure 4:**
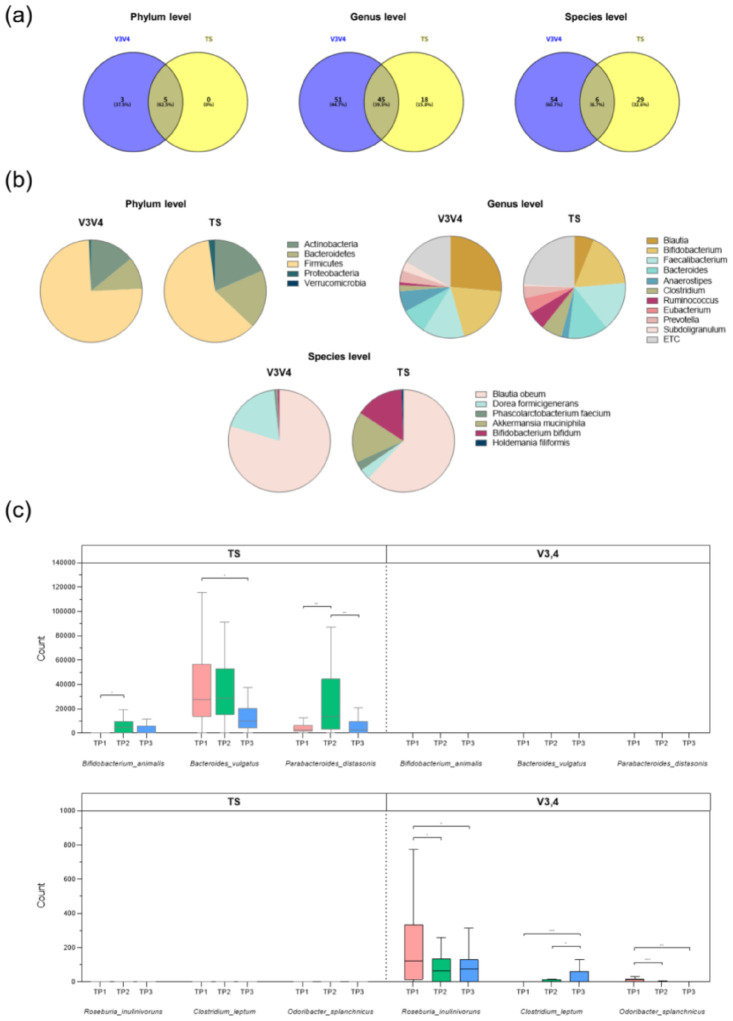
Comparative analysis of the taxonomic resolution and quantitative accuracy between 16S rRNA V3–V4 and targeted species sequencing (TSS). (**a**) Venn diagrams illustrating the number of common and unique taxa identified via the 16S V3–V4 and TSS methods at the phylum, genus, and species levels. The analysis highlights a significant drop in taxonomic consensus at lower levels, particularly at the species level, underscoring the critical failure of V3–V4 in achieving the necessary resolution for specific probiotic monitoring. (**b**) Pie or bar charts displaying the relative abundance profiles of key taxa commonly identified through both the V3–V4 and TSS methods (e.g., dominant phyla or the top 10 genera). This comparison shows the overall consensus in high-abundance community structures, contrasting with the performance of the method in classifying the microbial baseline. (**c**) Box plots comparing the relative abundance of discrepant and targeted low-abundance species across the three measured time points (T1–T3), with statistical differences assessed via ANOVA and Bonferroni post hoc analysis. The box plot ranges from the 25th–75th percentile, with the 50th percentile represented by the black horizontal line. The *p*-values for the Mann–Whitney U test are shown in the figure: * *p* < 0.05, ** *p* < 0.01 and *** *p* < 0.005. Abbreviation: TP, time point.

A critical comparative analysis between the 16S V3–V4 method and high-resolution TSS approach was performed to directly address limitations of the taxonomic resolution and quantification bias of V3–V4 (Figure 4). TSS analysis utilized a custom assay designed to target 73 specific bacterial species, thus providing the necessary fidelity to accurately track the administered probiotic strain. Detailed sequencing summaries, including total reads and total valid mapped reads obtained via the high-resolution TSS approach, are presented in Supplementary Data S2, along with a summary of the study subjects’ surveys.

#### 3.2.1. Taxonomic Resolution and Overlap Disparity

The comparative analysis, which used T2 (end of intervention) samples, revealed a progressive disparity in taxonomic classification between the two methods as the resolution increased (Figure 4a). At the highest level (phylum), the overlap was substantial, with five common taxa identified, representing 62.5% of all identified phyla. This agreement decreased at the genus level, with only 45 shared taxa, representing 39.5% of the total identified genera. Crucially, taxonomic consensus collapsed at the species level. Only six species were commonly identified through both the V3–V4 and TSS methods, constituting a mere 6.7% overlap with the total number of identified species. This severe divergence at the species level empirically validates the assertion that the V3–V4 method suffers from insufficient taxonomic resolution, making it unreliable for the precise species-level identification required for tracking specific strains. Comparisons of the relative abundance profiles of commonly identified taxa (Figure 4b) further demonstrated quantitative disagreement despite the qualitative consensus observed at taxonomic higher levels. For instance, the V3–V4 method reported *Blautia* abundance at 26.32% and that of *Bacteroides* at 8.19%, whereas TSS measured these at 6.22% and 12.56%, respectively. This substantial quantitative variance confirms that even when taxa are commonly identified, the underlying abundance data are fundamentally different between the two platforms.

#### 3.2.2. Quantitative Bias in Probiotic Strain Detection

Analysis of discrepant taxa across the measured time points provided critical evidence of the quantitative bias of V3–V4 sequencing, particularly concerning the administered probiotic strain (Figure 4c). Successful probiotic tracking was achieved when using the TSS method with species-specific primers, which enabled successful quantification of the administered probiotic. The *B. animalis* probiotic was detected and showed a temporal change consistent with the intervention, although the ANOVA post hoc result was marginally significant for T1 vs. T2 (*p* = 0.46). TSS also identified significant dynamic changes in other Bacteroidetes taxa: *Bacteroides vulgatus* showed a significant difference between T1 and T3 (*p* = 0.021), and *Parabacteroides distasonis* showed significant changes between T1 vs. T2 (*p* = 0.012) and T2 vs. T3 (*p* = 0.018). Contrastingly, V3–V4 quantification failed to measure the administered probiotic strain. While TS successfully identified and tracked *B. animalis* across the tested time points, V3–V4 reported no counts for this species, indicating the complete absence of detection. Furthermore, V3–V4 detected unique and statistically significant temporal changes in other species that were not found to be significant by TSS: *Roseburia inulinivorans* (T1 vs. T2, *p* = 0.03; T1 vs. T3, *p* = 0.04), *Clostridium leptum* (T1 vs. T3, *p* = 0.005; T2 vs. T3, *p* = 0.029), and *Odoribacter splanchnicus* (T1 vs. T2, *p* = 0.005; T1 vs. T3, *p* = 0.002). This discrepancy confirms that the V3–V4 method generates unique and statistically significant findings that are likely products of amplification bias rather than of true biological changes, thereby invalidating its reliability for efficacy assessments.

## 4. Discussion

In this longitudinal pilot study, we primarily employed standard 16S rRNA V3–V4 sequencing to screen the ecological impact that synbiotic intervention has on the gut microbiota of older adults. Our initial observations revealed a significant, albeit transient, reduction in alpha-diversity (Observed ASVs) during the intervention period (T2), suggesting that the administration of high-concentration probiotics induced a distinct “ecological perturbation” within the resident ecosystem [20,21,22,23]. Previous studies have posited that the influx of exogenous microorganisms can trigger temporary resource competition or antimicrobial activity, leading to the suppression of less competitive, rare taxa [24,25]. Consequently, the observed reduction in diversity should not be strictly interpreted as detrimental dysbiosis but rather as an indicator of active ecosystem remodeling and acute response of the community to probiotic intervention [20,26,27].

This complexity was further mirrored in the multidirectional shifts observed at the genus level. The V3–V4 analysis highlighted dynamic fluctuations, such as a significant decrease in dominant taxa (*Bacteroides*) and concurrent shifts in SCFA-producing genera, including *Roseburia* and *Odoribacter*. Furthermore, functional prediction analysis suggested a potential enhancement in the butanoate metabolism pathway during the intervention, suggesting that metabolic niche expansion was driven by the synbiotics [5,28]. However, these broad taxonomic and functional signals present a critical interpretative challenge; limited resolution of the V3–V4 region inherently fails to distinguish whether these shifts are directly driven by colonization of the administered strains or merely through compensatory responses of the resident species [29]. This “interpretative ambiguity” underscores the insufficiency of broad-spectrum profiling for elucidating the precise mechanisms of probiotic action [30].

The most definitive finding of this study, which empirically validates the methodological limitations raised in the Introduction, was the quantitative failure of the V3–V4 platform to detect the administered probiotic strain, *B. animalis* subsp. *lactis*. Furthermore, the superior accuracy of the TSS method was functionally validated through its ability to establish significant correlations with host clinical parameters, which were entirely masked in the 16S dataset. As presented in our correlation analysis (Supplementary Figure S1), the increase in *Bacteroides vulgatus*—precisely quantified only by TSS—was significantly associated with a reduction in Triglycerides (R = −0.627, *p* = 0.004) and CRP (R = −0.474, *p* = 0.041). This demonstrates that TSS not only mitigates technical bias but also provides the necessary quantitative resolution to evaluate the actual clinical efficacy of synbiotic interventions. Although the high-resolution TSS assay confirmed that *B. animalis* was the only species to exhibit a statistically significant increase at the T2 endpoint, the V3–V4 method reported near-zero abundance for this species across all measured time points. Although the high-resolution TSS assay confirmed that *B. animalis* was the only species to exhibit a statistically significant increase at the T2 endpoint, the V3–V4 method reported near-zero abundance for this species across all measured time points. This discrepancy is likely attributable to the well-documented primer mismatches in the V3–V4 region for Actinobacteria, particularly for the *Bifidobacterium* genus, which lead to severe amplification bias and false-negative results [11,31,32]. Moreover, the dramatic drop in taxonomic consensus at the species level, as evidenced by our Venn diagram analysis, confirms that the short read length of the V3–V4 region lacks the discriminatory power necessary to resolve closely related species [29,31]. Consequently, relying solely on standard 16S sequencing would have led to the erroneous conclusion that the probiotic intervention failed to colonize the host, thereby misrepresenting the clinical efficacy of the supplement [31].

The ultimate utility of our comparative analysis lies in resolving the functional ambiguity surrounding the V3–V4 findings. The TSS data provide the critical missing link: successful colonization of the administered *B. animalis* at T2 strongly supports the increased potential for butanoate metabolism, as *Bifidobacterium* species are primary producers of acetate and lactate, which are vital cross-feeding substrates for major butyrate producers, such as *Roseburia* and *Faecalibacterium* [33,34,35]. Furthermore, TSS identified significant T2 shifts in *Bacteroides vulgatus* and *P. distasonis*, key polysaccharide degraders, suggesting that the synbiotic induced functional reorganization of the dietary fiber-utilizing capacity within the gut [36,37,38]. This reorganization may play a crucial role in enhancing host adaptation and ecological resilience. According to Liu et al. (2025), gut microbiota significantly aids host adaptation under physiological or environmental stress through microbial-mediated mechanisms [39]. In the context of our study, the selective modulation of species such as *B. vulgatus* and *P. distasonis* via synbiotics may reinforce the metabolic homeostasis and resilience of the elderly host against age-related physiological declines. Conversely, the V3–V4 analysis yielded statistically significant changes in *R. inulinivorans*, *C. leptum*, and *O. splanchnicus* that were not corroborated by TSS, highlighting a methodological risk in which V3–V4 bias can generate statistically significant but potentially false signals from nontargeted species [32,40]. Finally, the V3–V4-predicted decreases in riboflavin metabolism and glycosphingolipid biosynthesis pathways during the intervention, although derived from ambiguous taxonomic data, may reflect a temporary suppression or metabolic shift in certain B-vitamin-producing Firmicutes taxa due to competitive influx of the *Bifidobacterium* strain, emphasizing the broad, cascading metabolic effects of the intervention [41,42].

Despite the significant methodological insights gained from this comparative analysis, several limitations of the present study must be acknowledged. First, as a pilot study, the sample size was relatively small (*n* = 20), which may limit the generalizability of the findings and statistical power to detect more subtle ecological shifts within the microbiota. Second, while the inclusion of participants on diabetic medication (*n* = 3) could be a potential confounding factor, our PERMANOVA analysis confirmed that medication use did not significantly alter the baseline microbial community structure (F = 2.04, R^2^ = 0.034, *p* = 0.060). This suggests that the observed ecological shifts were primarily driven by the synbiotic intervention rather than pharmacological variables. Third, our reliance on the V3–V4 region for broad community profiling remains a source of inherent technical constraint. The well-documented primer mismatches and limited taxonomic resolution of this region imply that some resident taxa might have been underrepresented or misidentified, potentially masking deeper interactions between the synbiotic and indigenous microbes of the host. Future large-scale longitudinal trials incorporating metagenomic shotgun sequencing alongside TSS assays would be beneficial to provide even greater functional depth and validate these findings across a more diverse population.

In conclusion, returning to the primary objective of the present study, we have empirically demonstrated that while standard 16S rRNA V3–V4 sequencing is a cost-effective tool for profiling overall community structure and detecting macroscopic ecosystem shifts, it is fundamentally inadequate for the precise monitoring of specific probiotic strains. The most critical finding of our comparative analysis is the quantitative bias stemming from intrinsic limitations of V3–V4 sequencing, which led to the outright non-detection of the administered *B. animalis* strain. This failure confirms that relying solely on V3–V4 sequencing risks misinterpreting the clinical efficacy of intervention trials. The rationale for utilizing the high-resolution TSS method is not only crucial for enhanced resolution but also to introduce a necessary quantitative truth standard into probiotic efficacy assessments. The TSS assay, designed with eight comprehensive 16S hypervariable regions and highly species-specific primers, inherently mitigates the two fatal flaws of the V3–V4 method: primer mismatch issues and low species-level fidelity. Successful quantification of the probiotic strain via TSS validated that this method provides unambiguous, species-level quantitative data that are essential for confirming strain survival and assessing the true ecological contribution of the supplement.

This research, which is positioned as a critical methodological validation study, establishes empirical data required to assess the potential of the V3–V4 method to misrepresent or miss changes in key functional taxa—including the administered probiotic species. To overcome these critical limitations and ensure the accurate validation of probiotic efficacy in future clinical research, we advocate the adoption of a dual-sequencing approach. By integrating the broad ecological screening capabilities of 16S rRNA V3–V4 sequencing with the precise, species-level quantification of TSS, researchers can ensure that both the “forest” (community dynamics) and “trees” (specific strain fate) are accurately assessed, thereby establishing the necessary scientific rigor in the field.

## Figures and Tables

**Table 1:** Characteristics of study subjects.

		Value (%)	
Total		20	
SEX			
	Male	3	(15)
	Female	17	(85)
Age			
	60s	8	(40)
	70s	11	(55)
	80s	1	(5)
	Range	64–83	
Smoking			
	no	19	(95)
	yes	1	(5)
Alcohol			
	no	14	(70)
	yes	6	(30)

**Table 2:** Predicted Functional Pathway Shifts by KEGG Analysis.

Pathway	Definition	*p*-Value	*p*-Value (FDR)	T1	T2	T3
ko04973	Carbohydrate digestion and absorption	0.0029	0.3485	0.0412	0.0554	0.0432
ko00604	Glycosphingolipid biosynthesis—ganglio series	0.0063	0.3485	0.0566	0.0405	0.0470
ko04016	MAPK signaling pathway—plant	0.0038	0.3485	0.0447	0.0505	0.0456
ko04626	Plant-pathogen interaction	0.0035	0.3485	0.1346	0.1130	0.1242
ko00020	Citrate cycle (TCA cycle)	0.0119	0.4404	0.4111	0.3851	0.4014
ko00650	Butanoate metabolism	0.0173	0.4788	0.4884	0.4659	0.4897
ko04724	Glutamatergic synapse	0.0200	0.5212	0.0879	0.0841	0.0872
ko00626	Naphthalene degradation	0.0063	0.3485	0.0516	0.0615	
ko05143	African trypanosomiasis	0.0063	0.3485	0.0162	0.0197	
ko01503	Cationic antimicrobial peptide (CAMP) resistance	0.0035	0.3485	0.3653	0.3033	
ko00740	Riboflavin metabolism	0.0049	0.3485	0.2751	0.2497	
ko00350	Tyrosine metabolism	0.0087	0.4279	0.1990	0.2234	
ko00051	Fructose and mannose metabolism	0.0149	0.4404	0.7792	0.7110	
ko01501	beta-Lactam resistance	0.0138	0.4404	0.4744	0.4186	
ko01040	Biosynthesis of unsaturated fatty acids	0.0149	0.4404	0.1225	0.1329	
ko00950	Isoquinoline alkaloid biosynthesis	0.0110	0.4404	0.0582	0.0723	
ko01052	Type I polyketide structures	0.0128	0.4404	0.0195	0.0251	
ko04950	Maturity onset diabetes of the young	0.0215	0.5289	0.0006	0.0009	
ko00945	Stilbenoid, diarylheptanoid and gingerol biosynthesis	0.0231	0.5380	0.0160	0.0134	
ko00980	Metabolism of xenobiotics by cytochrome P450	0.0373	0.6490	0.0604	0.0683	
ko00791	Atrazine degradation	0.0425	0.6490	0.0365	0.0420	
ko00513	Various types of N-glycan biosynthesis	0.0425	0.6490	0.0673	0.0537	
ko00511	Other glycan degradation	0.0349	0.6490	0.3629	0.2941	
ko03008	Ribosome biogenesis in eukaryotes	0.0398	0.6490	0.0463	0.0542	
ko03420	Nucleotide excision repair	0.0425	0.6490	0.3649	0.3854	
ko00720	Carbon fixation pathways in prokaryotes	0.0305	0.6490	0.6769	0.6423	
ko00190	Oxidative phosphorylation	0.0349	0.6490	0.7969	0.7601	
ko04075	Plant hormone signal transduction	0.0398	0.6490	0.0109	0.0077	
ko00790	Folate biosynthesis	0.0398	0.6490	0.4522	0.4404	
ko03018	RNA degradation	0.0483	0.7133	0.4481	0.4251	
ko03450	Non-homologous end-joining	0.0349	0.9851	0.0033		0.0044
ko01054	Nonribosomal peptide structures	0.0483	0.9851	0.0114		0.0125
ko00533	Glycosaminoglycan biosynthesis—keratan sulfate	0.0349	1.0000		0.0005	0.0008
ko01523	Antifolate resistance	0.0149	1.0000		0.1638	0.1582
ko00904	Diterpenoid biosynthesis	0.0231	1.0000		0.0001	0.0001
ko00640	Propanoate metabolism	0.0231	1.0000		0.5076	0.5235

## Data Availability

The original contributions presented in this study are included in the article/supplementary material. Further inquiries can be directed to the corresponding authors.

## Supplementary Materials

The following supporting information can be downloaded at: https://www.mdpi.com/article/10.3390/microorganisms14051035/s1, Figure S1: Correlation analysis between TSS-detected microbial abundance and clinical metabolic parameters. Supplementary Data S1: brief description; Table S1: species-specific primers targeting 73 bacterial species; Table S2: Summary of study subjects surveys.

## Abbreviations

The following abbreviations are used in this manuscript:

ANOSIM	Analysis of Similarities
ANOVA	Analysis of variance
ASV	Amplicon sequence variant
CFUs	Colony-forming units
NMDS	Nonmetric multidimensional scaling
QIIME	Quantitative Insights into Microbial Ecology
SCFA	Short-chain fatty acid
TSS	Targeted species sequencing
