# Supplementary figures and images for: Probiotic-Induced Gut Microbiota Modulation: A Comparative Analysis Using 16S rRNA V3–V4 and Targeted Sequencing
Source: Microorganisms. 2026 May 1;14(5):1035. doi: 10.3390/microorganisms14051035 (PMC13210024; doi:10.3390/microorganisms14051035)

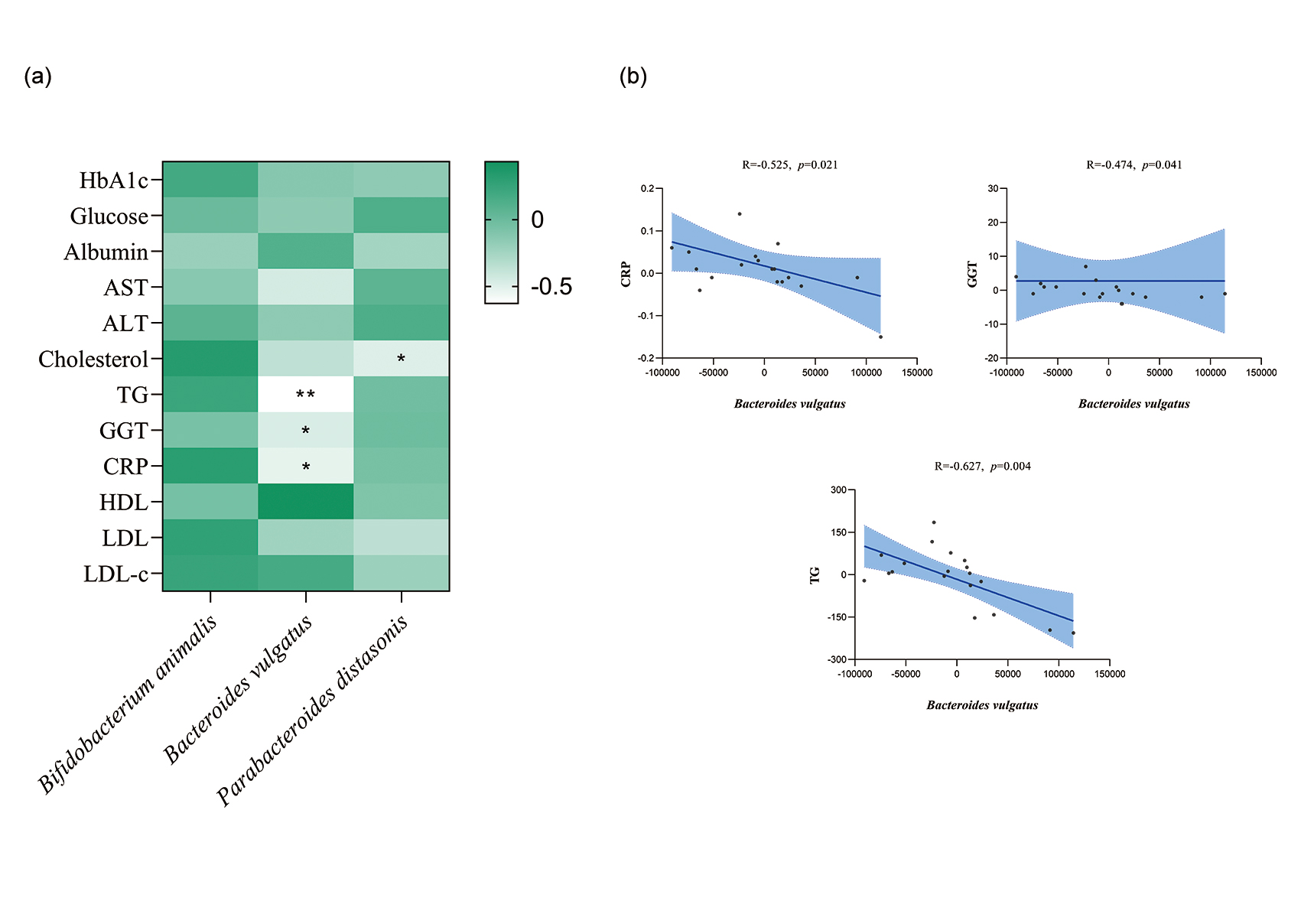

Supplement: Supplementary file 1 [file microorganisms-14-01035-s001.zip › Supplementary Fig1.jpg]
